# Children’s Block-Building Skills and Mother-Child Block-Building Interactions Across Four U.S. Ethnic Groups

**DOI:** 10.3389/fpsyg.2019.01626

**Published:** 2019-07-12

**Authors:** Daniel D. Suh, Eva Liang, Florrie Fei-Yin Ng, Catherine S. Tamis-LeMonda

**Affiliations:** ^1^Center for Research on Culture, Development, and Education, Department of Applied Psychology, New York University, New York, NY, United States; ^2^Department of Educational Psychology, The Chinese University of Hong Kong, Sha Tin, Hong Kong

**Keywords:** spatial skills, spatial cognition, STEM learning, ethnic minorities, block building

## Abstract

Play offers an unparalleled opportunity for young children to gain cognitive skills in informal settings. Block play in particular—including interactions with parents around block constructions—teaches children about intrinsic spatial features of objects (size, shape) and extrinsic spatial relations. In turn, early spatial cognition paves the way for later competencies in math and science. We assessed 4- and 5-year-old children’s spatial skill on a set of block-building constructions and examined mother-child block building interactions in 167 U.S. dyads from African American, Dominican, Mexican, and Chinese backgrounds. At both ages, children were instructed to copy several 3D block constructions, followed by a “break” during which mothers and children were left alone with the blocks. A form that contained pictures of test items was left on the table. Video-recordings of mother-child interactions during the break were coded for two types of building behaviors – test-specific construction (building structures on the test form) or free-form construction (building structures not on the test form). Chinese children outperformed Mexican, African American, and Dominican children on the block-building assessment. Further, Chinese and Mexican mother-child dyads spent more time building test-specific constructions than did African American and Dominican dyads. At an individual level, mothers’ time spent building test-specific constructions at the 4-year (but not 5-year) assessment, but not mothers’ initiation of block building interactions or verbal instructions, related to children’s performance, when controlling for ethnicity. Ethnic differences in children’s block-building performance and experiences emerge prior to formal schooling and provide a valuable window into sources of individual differences in early spatial cognition.

## Introduction

Spatial cognitive skills involve perceiving spatial information, such as object shape and relative location, and mentally and/or physically manipulating objects in space. Spatial skills are foundational to later success in Science, Technology, Engineering, and Mathematics (STEM) subjects and careers (Caldera et al., 1999; Assel et al., 2003; Chen, 2009; Wai et al., 2009; Uttal and Cohen, 2012; Lombardi et al., 2017). Consequently, interest in the early development of spatial skills has grown. Indeed, variation in preschoolers’ and even infants’ spatial skills relates to later math and spatial cognition (Lauer and Lourenco, 2016; Verdine et al., 2017).

Everyday play with blocks provides children with valuable opportunities to acquire spatial cognitive skills in informal settings, well before formal exposure to science and math subjects. During block building, children perceive and learn about intrinsic features of objects, such as how objects vary along dimensions of size, pattern, symmetry, and shape (Casey and Bobb, 2003; Verdine et al., 2014). Furthermore, block play supports children’s representations of extrinsic spatial relations (e.g., in, behind; Reifel, 1984) and mental rotation skills (Wexler et al., 1998) because children actively manipulate spatial relations by aligning and rotating blocks and placing them on top of or next to one another. Parent-child block building can further promote children’s spatial skill development through hands-on and verbal guidance (Lombardi et al., 2017; Borriello and Liben, 2018) and spatial language (Ferrara et al., 2011; Pruden et al., 2011), which facilitate children’s attention to spatial concepts and aid spatial learning.

Block building is not only a vehicle for children to develop spatial skills, but block-building assessments that require children to copy specific block constructions have been shown to reliably index children’s spatial skill and predict later STEM performance, including mathematics (Verdine et al., 2014, 2017).

In light of the importance of block building as an activity that promotes spatial skill and a window into young children’s spatial skill performance, we tested U.S. children from African American, Dominican, Mexican, and Chinese backgrounds on a set of block constructions and investigated mothers’ spontaneous interactions with children around block building. We tested children from diverse ethnic backgrounds because of longstanding differences in later STEM performance. By observing children separately and together with their mothers, we asked whether ethnic differences exist in children’s block-building performance early in development and if so, whether ethnic differences relate to parent-child block-building interactions.

### Block Building and Parental Supports

Block building offers children rich opportunities to learn and practice spatial skills, and block building with parents might further scaffold children’s spatial skill development. Parents have been shown to use gestures and teach children efficient spatial strategies during block building interactions (Lombardi et al., 2017). Block building also elicits parent spatial language, which relates to children’s spatial language and spatial skill (Pruden et al., 2011; Miller et al., 2017). In fact, playing with blocks elicits more spatial language from parents than other everyday activities, such as drawing, playing house, dressing up, throwing a ball, or playing with animal figurines or food and kitchen toy sets (Ferrara et al., 2011). Furthermore, dyadic block-building activities that center around constructing structures from pictures prompt even more parent spatial language than free-form block construction (Ferrara et al., 2011; Borriello and Liben, 2018). Thus, differences in mother-child block building may contribute to individual and ethnic differences in children’s block building and spatial skill.

### Research Gaps: Ethnic Differences in Block Building and Parental Supports

Ethnic differences in STEM are well-documented. Asian students receive higher standardized test scores and average grades in STEM high school subjects (Reardon, 2008; Nord et al., 2011) and are twice as likely as their Black and Latino counterparts to obtain degrees in STEM fields (Chen, 2009). The 2011 National Assessment of Educational Progress (NAEP) math assessment revealed that 4th and 8th grade Asian students score higher than Black and Latino students (Gonzalez and Kuenzi, 2012). Even by school entry, Asian kindergarteners’ math performance is higher than that of Black and Latino kindergarteners (Sonnenschein and Sun, 2017).

However, ethnic differences in children’s block-building performance and parent-child block-building interactions remain largely unexplored, although these skills and interactions may be foundational to children’s later STEM performance. A greater percentage of Chinese than Latino 4- to 6-year-olds in the United States engaged in block building at home at least once a week (56.4 vs. 45.9%; Sonnenschein et al., 2018). In contrast, when Black, Latino, and Asian parents were asked how often their children played with blocks, although in the context of many other activities, no differences were found (Sonnenschein and Sun, 2017). Thus, whether ethnic differences exist between Black, Chinese and Latino children in block-building performance and parent-child block-building behaviors remains relatively unexplored.

Differences in parent practices and involvement in other domains hint at potential ethnic differences around block building as well. Chinese mothers are explicit and systematic about teaching their children at home (Huntsinger et al., 2000), and use concrete expectations and plans to promote children’s learning (Sonnenschein et al., 2018). Therefore, Chinese mothers may intentionally allot time for block building and provide support for block-building activities and spatial skill development. Alternatively, Chinese mothers may only consider formal, practice-oriented (e.g., workbooks) activities as educational (Huntsinger and Jose, 2009). If so, they may be unlikely to engage with their children during block building.

### Current Study

We examined 4- and 5-year-old children’s spatial skills and interactions with mothers around block building. We included U.S. dyads from African American, Dominican, Mexican, and Chinese backgrounds to extend beyond the dominant focus on European-American dyads (e.g., Ferrara et al., 2011; Lombardi et al., 2017; Borriello and Liben, 2018). Three aims guided this study.

First, we examined within- and between-group ethnic differences in 4- and 5-year-olds’ block-building performance. We tested children’s ability to replicate a set of structures an experimenter built as children watched. We asked whether ethnic differences in spatial skills around block building exist already by 4 and 5 years of age. We were uncertain about the patterns we might obtain. One possibility is that children at young ages, prior to the onset of formal schooling, do not differ in their block-building performance because within-group variation swamps between-group differences. Alternatively, Chinese children may surpass children of Latino and African American backgrounds already by 4 years of age, or at least by the time they reach 5 years, thereby aligning with ethnic and racial differences in STEM that have been documented in school-aged children.

Second, we investigated whether mothers and children from different ethnicities differ in their block-building interactions. To address this aim, we left dyads alone in a room with blocks without instructions, to reduce social desirability and pressure on mothers to encourage children’s block building or build with their children. We left a sheet of images of test structures on the table and visible to dyads. Based on previous findings that Chinese parents are more intentional about teaching their children (Huntsinger et al., 2000; Sonnenschein et al., 2018), we expected Chinese mothers and children to engage in more block building overall, especially test-specific constructions. Furthermore, we expected Chinese mothers to initiate interactions and provide instruction around block building more than Latino and African American mothers because Chinese mothers may be most likely to view dyadic block building as a teaching opportunity. We also expected mothers’ and children’s building behaviors during the interaction to covary, such that if mothers engaged in test-specific constructions, children would do so, and if mothers engaged in free-form constructions, children would do the same.

Third, we examined associations between mother-child block construction behaviors and independent assessments of children’s block-building performance. Do mothers’ behaviors during block-building interactions relate to children’s block-building skill? We expected mothers who provide high instructional support and hands-on guidance during block building to have children with high performance in block building.

## Materials and Methods

### Participants

Participants were 167 African American (*n* = 36), Dominican (*n* = 43), Chinese (*n* = 51), and Mexican (*n* = 37) mothers and their children (83 boys, 84 girls) recruited from hospitals and clinics in the New York City metropolitan area. Criteria for participation included: (1) mother being at least 18 years old at the time of her child’s birth, (2) child being healthy and full term at birth, and (3) child living with mother since birth. African American mothers were predominantly fourth generation immigrants (61.1%) and Dominican mothers were first (72.1%) and second (27.9%) generation immigrants. Chinese and Mexican mothers were the more recent immigrant groups with 100% being first generation. African American and Dominican mothers completed an average of 12.03 (*SD* = 1.38) and 12.57 (*SD* = 2.06) years of formal education, respectively. Chinese mothers completed an average of 10.94 (*SD* = 2.80) years of formal education, whereas Mexican mothers completed the fewest years of formal education with an average of 7.97 (*SD* = 3.50) years. In addition, 63.5% of the 4-year-old children were in Pre-K at the time of their participation and by the time children were 5 years of age, 84.4% were in kindergarten. We obtained written informed consent from participants, parental consent for children, and signed consent to share videos on Databrary.org, an online open data-sharing platform for researchers to access video data.

Mothers and children visited our lab when children (*N* = 167) were age 4 (*M* = 4.20, *SD* = 0.15) and 5 years (*M* = 5.15, *SD* = 0.15). At each age, children engaged in a block-construction assessment that was developed by the third author, during which children were required to replicate 3D block constructions that were built by the experimenter as children watched. The assessment was followed by a 5-minute “break” where the mother-child dyad could play with the blocks. A video camera recorded children’s performance and mother-child block-building behaviors during the break.

#### Block-Building Assessment

The experimenter presented the child with two identical sets of differently colored blocks (red and blue) that contained all the pieces required to construct the assessment items. The child was allowed to choose which set of blocks to use, and the experimenter used the other set of blocks. The experimenter then built a sample block construction before beginning the assessment and asked the child to build the same construction immediately following. The first easy pretest item ensured the child understood the task before proceeding with the actual assessment.

**Figure 1:**
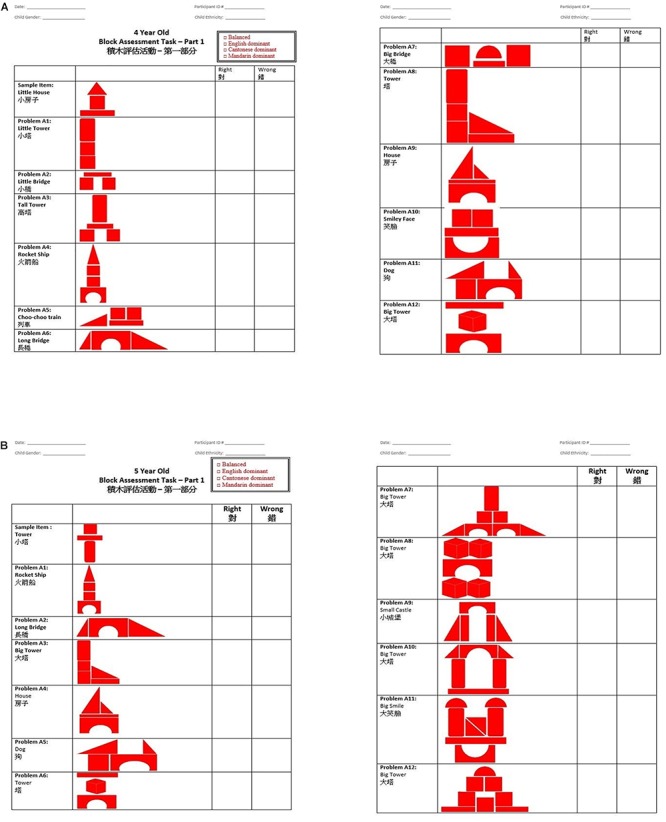
Block-building assessment scoring sheets at the **(A)** 4-year assessment, and **(B)** 5-year assessment.

The experimenter then continued with the block construction assessment, first demonstrating how to build each block construction with her set of blocks and then asking the child to replicate the construction with his or her blocks. Children were tested on a set of 12 test-items of increasing difficulty (Figure 1A,B). The experimenter marked down the child’s performance on a scoring sheet, and proceeded to the next item. If the child received three consecutive items incorrect or completed all assessment items, the test ended. Children’s performance was indicated by the number of items they built correctly. Test items for the 4- and 5-year assessment were tested in a pilot study and deemed to be appropriate at each age and for all ethnic groups.

#### Mother-Child Block Building

After the assessment, mothers were told that children would have a short 5-minute “break.” The experimenter stated that “(*Child*’*s name*) *is going to have a short break now and I thought it*’*d be nice for you to join him/her while I go get some things done in the other room*.” We chose not to directly ask mothers to play with their children to reduce demand characteristics and to maximize variability. This low-demand situation was thought to better capture what might occur in a natural home environment.

Both sets of blocks were left on the table between mothers and children. Additionally, the scoring sheet that contained pictures of the test-specific constructions was left on the table. The experimenter then left the mother and child for 5 min with the camera recording. Mothers and their children were unaware that they were being video-recorded. After the 5-minute mother-child “break,” the experimenter returned and continued with a different assessment.

### Coding of Mother-Child Interactions

The video-recorded mother-child block construction break was coded using INTERACT Software (Mangold, 2015). Of the 5-minute break, 4 min were coded, starting when mother sat down next to the child. The full 5 min were not coded because dyads differed in the amount of time they took to settle down at the table. From videos, we coded the degree to which the mother or child led the block-building interactions; how much hands-on time child and mother spent building with the blocks; and mothers’ verbal instruction around block building.

The degree to which mother or child led in the block building (termed *initiation*) was coded on a 5-point Likert scale (1 = Child initiates and engages in building >90% of the time; 2 = Child initiates and engages in building 70–90% of the time; 3 = Child and mother equally initiate building; 4 = Mother initiates and engages in building 70–90% of the time; 5 = Mother initiates and engages in building >90% of the time.). Coding of initiation yielded a single score for the interaction.

Children’s and mothers’ time spent block building were coded separately based on the total duration (in seconds) each person spent actively building. The onset of a block building bout was defined by touching and moving a block and ended when the child or mother stopped touching and moving a block. We further analyzed time spent building into two types of construction activities: test-specific construction and free-form construction. *Test-specific construction* was coded when mothers and/or children built a test item on the scoring sheet. Mothers and children were considered as building a test-specific item if they referred to the scoring sheet and built something that looked exactly like or similar to an item on the scoring sheet (mistakes were allowed). This included time spent disassembling the item after it was built. *Free-form construction* was coded when mothers and/or children built something with the blocks other than the test items. Mothers’ *Verbal Instruction* on how to build with the blocks was coded using a time sampling approach. The block-building interaction was divided into 10-second intervals and coders marked each interval on whether mothers offered instructions around building to the child or not. Ten percent of videos were randomly selected and coded for inter-observer reliabilities. Kappa coefficients for measures ranged from 0.80 to 0.92.

## Results

Neither gender, preschool status, nor mother education related to mother or child block building. Therefore, models collapse across these variables.

### Individual and Ethnic Differences in Children’s Performance

**Figure 2:**
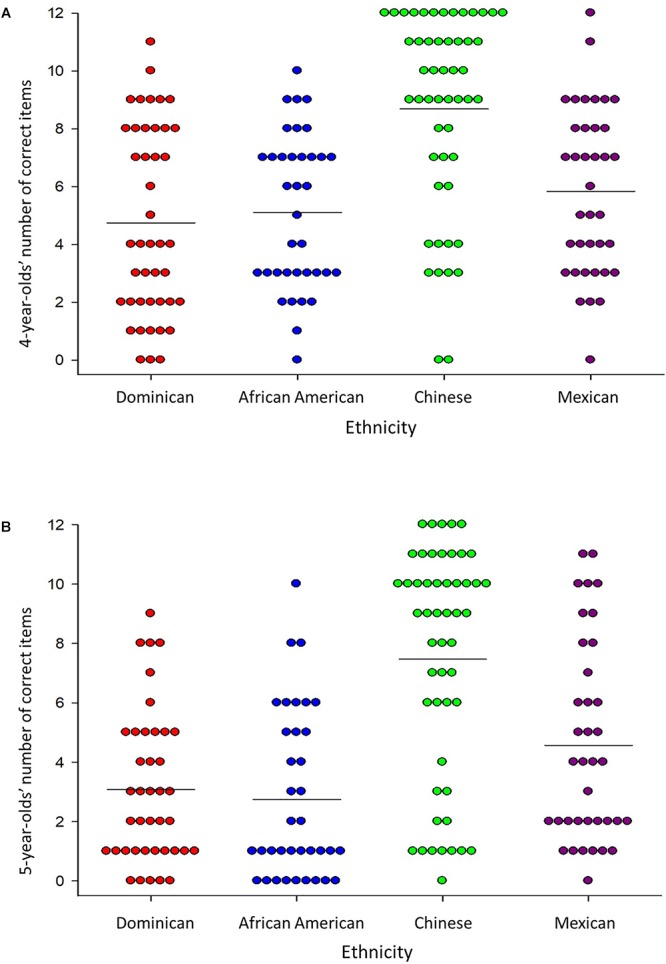
Number of correct items for children from each ethnic group at the **(A)** 4-year assessment, and **(B)** 5-year assessment. Each dot represents a child, and horizontal lines denote averages.

Children’s performance on block building at ages 4 and 5 years is displayed in Figure 2A,B. At both ages, children of all ethnicities varied substantially in their performance—ranging from 0 items correct to the maximum of 12 items correct.

To test ethnic differences in children’s performance, we conducted a 4 (Ethnicity) × 2 (Child Age) MANOVA, with the total number of correct items at each age serving as dependent variables. As hypothesized, Chinese children exceeded Mexican, Dominican, and African American children (all *p*’s < 0.05), as indicated by a main effect for Ethnicity, *F*(3,163) = 23.41*, p* < 0.001. This pattern maintained at both ages, although Mexican children outperformed African American children by age 5 years, *p* = 0.022. The Age × Ethnicity interaction was not significant, *F*(3,163) = 0.97, *p* = 0.41. Because difficulty of test items increased at the 5-year assessment, we did not examine age-related changes.

### Individual and Ethnic Differences in Mother-Child Block-Building Activities

#### Initiation

At both ages, mothers and children were balanced in leading the block-building interaction, as seen in the normal distribution around the mid-point of the 5-point scale (*M* = 3.18, *SD* = 1.05 and *M* = 3.20, *SD* = 1.12 at 4- and 5-year assessments, respectively). At the 4-year assessment, 39.4% of parent-child dyads were balanced on initiation (scores of 3); children led sometimes or all the time in 23.1% of dyads (scores of 1 and 2); and mothers led sometimes or all the time in 37.5% of dyads (scores of 4 and 5). At the 5-year assessment, 34% of parent-child dyads showed balance, 25.6% had children leading all the time or sometimes, and the remaining 40.4% were characterized by mother leading. A 4 (Ethnicity) × 2 (Child Age) MANOVA indicated no ethnic or age differences, as seen in non-significant main effects of Ethnicity, *F*(3,145) = 0.56, *p* = 0.65, and Age, *F*(1,145) = 0.002, *p* = 0.96. The Ethnicity × Age interaction was also not significant, *F*(3,145) = 1.06, *p* = 0.37. Thus, distribution of initiation ratings replicated across age and the four ethnicities.

#### Mothers’ Block Building

**Figure 3:**
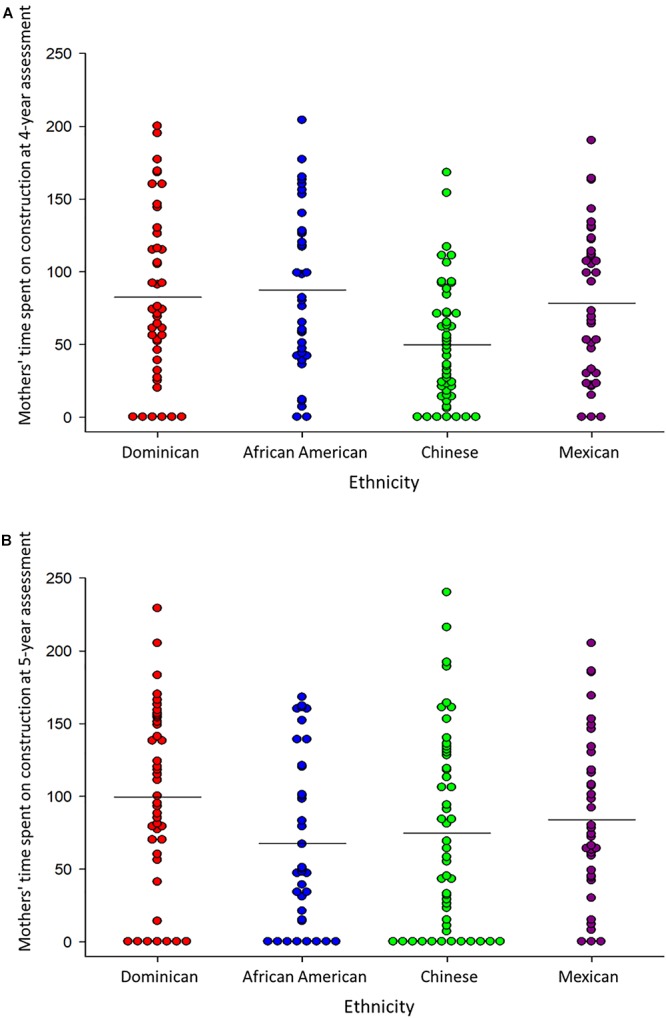
Overall time spent on construction activities by mothers from each ethnic group at the **(A)** 4-year assessment, and **(B)** 5-year assessment. Each dot represents a mother, and horizontal lines denote averages.

Mothers varied in the time they spent building with their children during the break, ranging from 0 to 204 s. A minority of mothers did not engage in any construction activities at the 4-year assessment (10.8%) and 5-year assessment (18.0%). Figure 3A,B display individual mothers’ construction activities at the two child ages.

We tested ethnic differences in mothers’ overall time in block building in a 4 (Ethnicity) × 2 (Child’s Age) MANOVA. Counter to hypotheses, Chinese mothers spent significantly *less time* building than did Dominican mothers collapsing across the two ages, as revealed in an Ethnicity main effect, *F*(3,163) = 3.74, *p* = 0.012. An Ethnicity × Age interaction, *F*(3,163) = 2.67, *p* = 0.049, revealed that when children were age 4, Chinese mothers spent less time building than all other mothers, *p*’s < 0.02. However, when children were 5 years of age, Chinese mothers were the only group to increase time spent on building, and consequently no longer differed from the other mothers, *p*’s > 0.05. African American mothers spent significantly less time than Dominican mothers in overall building when children were 5 years of age, *p* = 0.024.

Most centrally, we tested age and ethnic differences in the two types of mothers’ construction activities in a 4 (Ethnicity) × 2 (Construction Type: test-specific vs. free-form) × 2 (Child’s Age) MANOVA. Mothers spent more time on free-form construction than test-specific construction overall, *F*(1,163) = 11.60, *p* = 0.001. However, mothers of the 4 ethnicities differed in how they distributed time between the two construction types, as seen in a Construction Type × Ethnicity interaction, *F*(3,163) = 9.35, *p* < 0.001. African American and Dominican mothers spent more time building free-form structures than test-specific structures, *p*’s < 0.001, and spent more time on free-form construction than Mexican and Chinese mothers collapsing across the two ages, *p*’s < 0.01, although African American mothers decreased their time on free-form construction over child age, *p* = 0.021.

In contrast, Mexican mothers spent more time building test-specific structures than free-form structures, *p* = 0.05, and exceeded mothers of the other ethnicities on this type of construction, all *p*’s < 0.01. Further, Mexican mothers increased their time spent on test-specific structures between the two ages, *p* = 0.009. Like Mexican mothers, Chinese mothers engaged in more test-specific structures than free-form structures with their 4-year-olds; although, they built more free-form structures when children were 5 years of age, *p* = 0.032. Ethnic differences in patterns of change were confirmed in a 3-way Ethnicity × Construction Type × Child Age interaction, *F*(3,163) = 4.31, *p* = 0.006.

#### Children’s Block Building

**Figure 4:**
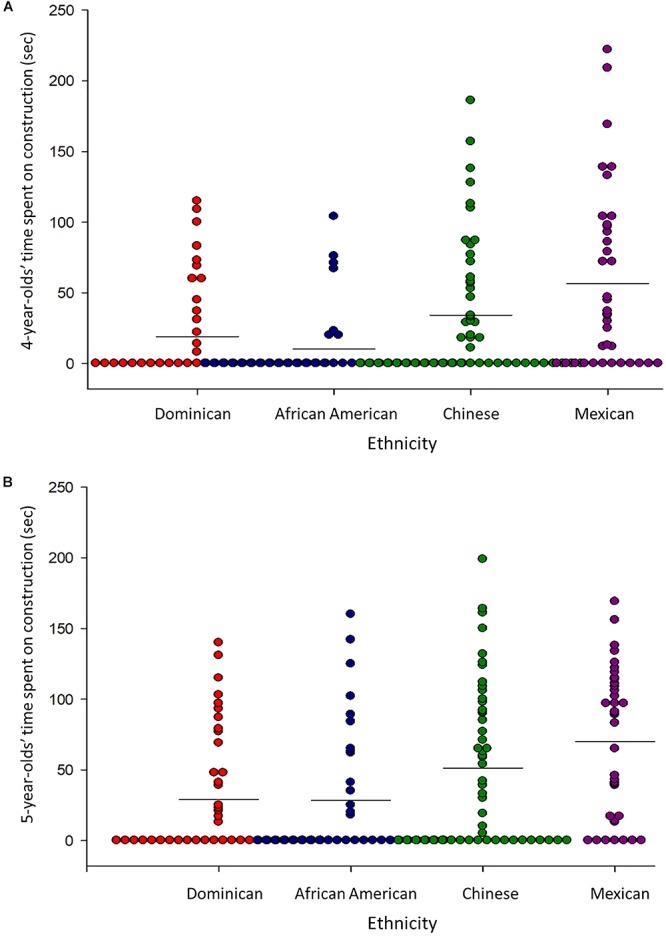
Overall time spent on construction activities by children from each ethnic group at the **(A)** 4-year assessment, and **(B)** 5-year assessment. Each dot represents a child, and horizontal lines denote averages.

Figure 4A,B display individual children’s construction activities at the two ages. Children, varied dramatically in their time spent building, ranging from 0 to 240 s.

Ethnic differences in children’s overall construction was tested in a 4 (Ethnicity) × 2 (Child’s Age) MANOVA. Children of the four ethnic groups marginally differed in their overall block building across both ages, *F*(3,163) = 2.59, *p* = 0.055. Overall, African American children spent significantly less time building than did Dominican and Chinese children, and Mexican children spent less time building than did Chinese children, all *p*’s < 0.05. An Ethnicity × Age interaction, *F*(3,163) = 3.02, *p* = 0.032, revealed that although ethnic differences were not seen at the 4-year assessment, *F*(3,163) = 0.71, *p* = 0.55, ethnic differences emerged by the 5-year assessment, *F*(3,163) = 4.94, *p* = 0.003. Like their mothers, Chinese children were the only group to increase time spent on block building between the two ages, *p* = 0.005.

We further tested age and ethnic differences in the two types of children’s constructions in a 4 (Ethnicity) × 2 (Construction Type) × 2 (Child’s Age) MANOVA. Paralleling the behaviors of mothers, children spent more time building free-form structures than test-specific structures overall, *F*(1,163) = 34.07, *p* < 0.001, but increased in test-specific structures between the two ages, Age × Construction Type, *F*(1,163) = 7.06, *p* = 0.009.

Children of the four ethnicities differed in how they distributed their time across the two construction types, with patterns mirroring those seen in mothers, as revealed by a 2-way Construction Type × Ethnicity interaction, *F*(3,163) = 11.43, *p* < 0.001. Like their mothers, Mexican (*p’*s < 0.01) and Chinese children (*p’*s < 0.05) spent more time building test-specific structures compared to Dominican and African American children, and Mexican children specifically spent more time on test-specific structures than free-form structures overall, *p*’s < 0.01. Reciprocally, Dominican children spent more time building free-form structures than Mexican and Chinese children, *p*’s < 0.05, but did not differ from African American children on this type of construction. The 3-way interaction was not significant, *F*(3,163) = 1.09, *p* = 0.354.

#### Mothers’ Verbal Instructions

Mothers varied in how often they verbally instructed children around block building, ranging from 0 to 24 intervals (*M* = 3.06, *SD* = 4.12 and *M* = 4.10, *SD* = 5.98, at 4- and 5-year assessments, respectively). Ethnic differences in mothers’ instruction was tested in a 4 (Ethnicity) × 2 (Child’s Age) MANOVA. Mothers of the four ethnic groups differed on their instruction, as seen by a main effect of Ethnicity, *F*(3,163) = 30.32, *p* < 0.001. Again, counter to hypotheses, Chinese mothers provided *less instruction* to their children (*M* = 0.91, *SE* = 0.46) than did Dominican (*M* = 4.22, *SE* = 0.50) and Mexican (*M* = 7.45, *SE* = 0.54*)* mothers when collapsing across ages, *p*’s < 0.001, and marginally less instruction than African American mothers (*M* = 2.63, *SE* = 0.54), *p* = 0.10.

Mexican mothers provided their children with the *most instruction* compared to African American, Dominican, and Chinese mothers, *p*’s < 0.001. Furthermore, an Ethnicity × Age interaction, *F*(3,163) = 6.96, *p* < 0.001, revealed that Mexican mothers were the only group to significantly increase their instruction to children from the 4-year assessment (*M* = 4.97, *SD* = 4.96) to the 5-year assessment (*M* = 9.92, *SD* = 7.84), *p* < 0.001. The increase in Mexican mothers’ instruction was confirmed in a main effect of Age, *F*(1,163) = 6.88, *p* = 0.01.

### Mother-Child Associations During Block Building

We next examined associations between mothers’ and children’s behaviors during block building, with focus on initiation, instruction, and the two forms of block building (test-specific and free-form structures).

#### Initiation and Child Block Building

At the 4-year assessment, high initiation, representing mothers leading the block-building interaction, did not relate to children’s time spent on test-specific construction, *r* = 0.13, *p* = 0.10, or free-form construction, *r* = -0.076, *p* = 0.34. However, when associations between initiation and children’s building were investigated by ethnicity, initiation related to children’s time spent building test-specific items for Dominican, *r* = 0.35, *p* = 0.028 and African American children, *r* = 0.53, *p* = 0.001, at the 4-year assessment. At the 5-year assessment, mothers’ initiation of block building related to children’s time spent building test-specific structures, *r* = 0.17, *p* = 0.039, and negatively related to children’s time spent building free-form structures, *r* = -0.21, *p* = 0.01. However, both associations were only seen in Chinese dyads, *r* = 0.52, *p* < 0.001, and *r* = -0.44, *p* = 0.002.

#### Mother Construction Type and Child Construction Type

As hypothesized, mothers’ and children’s block-building activities correlated in specific ways at both ages. Mothers’ time spent building free-form structures related to children’s time spent building free-form structures at the 4-year assessment, *r* = 0.52, *p* < 0.001, and 5-year assessment, *r* = 0.65, *p* < 0.001. Similarly, mothers’ time spent building test-specific structures related to children’s time building test-specific structures at the 4-year assessment, *r* = 0.57, *p* < 0.001, and the 5-year assessment, *r* = 0.56, *p* < 0.001. Associations were consistent and significant across all four ethnicities. At both assessments, mothers’ time spent building free-form structures related inversely to children’s time spent building test-specific structures, just as mothers’ time spent building test-specific structures related inversely to children’s time spent building free-form structures.

#### Instruction and Child Block Building

Instruction by mothers related to children’s time spent building test-specific structures at the 4-year assessment, *r* = 0.34, *p* < 0.001. This association was seen across Dominican children, *r* = 0.46, *p* = 0.002, African American children, *r* = 0.43, *p* = 0.009, Chinese children, *r* = 0.35, *p* = 0.01, and Mexican children (marginally), *r* = 0.29, *p* = 0.08. Similarly, at the 5-year assessment, Instruction related to children’s time spent building test-specific structures, *r* = 0.39, *p* < 0.001. This association again maintained across Dominican children, *r* = 0.49, *p* = 0.001, African American children, *r* = 0.62, *p* < 0.001, and Mexican children, *r* = 0.39, *p* = 0.018, and Chinese children (marginally), *r* = 0.26, *p* = 0.069.

### Associations Between Block-Building Interactions and Child Performance

Regressions next tested associations between the independent variables of mothers’ initiation, instruction, test-specific construction, and free-form construction in relation to children’s performance during the independent block-building assessment at each assessment age (Table 1). Ethnicity variables (with Chinese as referent group) were included in each model. The independent variables explained 26.3% of the variance in children’s block-building performance at the 4-year assessment, *R*^2^ = 26.3, *F*(7,152) = 7.76, *p* < 0.001. African American, Dominican, and Mexican ethnicity status negatively related to children’s block-building performance compared to the Chinese reference group, *B* = -0.34 to -0.43, *p*’s < 0.001. Furthermore, mothers’ time spent building test-specific structures related positively with children’s block-building performance when holding other independent variables constant, *B* = 0.17, *p* = 0.038. In contrast, neither initiation, *B* = -0.11, *p* = 0.16, nor mother’s instruction, *B* = -0.13, *p* = 0.13, related to child performance. For the 5-year assessment, independent variables accounted for 24.2% of the variance in children’s block-building performance, *R*^2^ = 24.2, *F*(7,148) = 6.76, *p* < 0.001. Ethnicity variables were significant for the African American group, *B* = -0.46, *p* < 0.001, and Dominican group, *B* = -0.37, *p* < 0.001 (but not Mexican, *B* = -0.19, *p* = 0.067), relative to the Chinese referent group at the 5-year assessment. By the 5-year assessment, mother’s time spent building test-specific structures no longer related to children’s performance, *B* = 0.023, *p* = 0.78, nor did initiation or instruction.

**Table 1:** Summary of Multiple Linear Regressions Analyses for Variables Predicting Children’s Performance.

	4 years of age (*n* = 160)			5 years of age (*n* = 156)		
Variable	*B*	*SE B*	*β*	*B*	*SE B*	*β*
Constant	9.45	0.87	–	7.88	0.97	–
Dummy coding of the African American group	-2.92	0.73	-0.35***	-4.36	0.79	-0.46***
Dummy coding of the Dominican group	-3.32	0.70	-0.41***	-3.21	0.78	-0.37***
Dummy coding of the Mexican group	-2.51	0.73	-0.30***	-1.74	0.94	-0.19^†^
Mother building test-specific items	0.02	0.01	0.17***	0.00	0.01	0.02
Mother building free-form items	-0.00	0.01	-0.05*	-0.00	0.01	-0.05
Verbal Instructions	-0.11	0.07	-0.13	-0.11	0.06	-0.17^†^
Initiation	-0.36	0.25	-0.11	-0.12	0.25	-0.04
*R^2^*		0.26			0.24	
*F*		7.76***			6.76***

^∗^*p < 0.05; ^∗∗∗^*p* < 0.001; ^†^*p* < 0.10*.

## Discussion

Informal opportunities to play with blocks arm children with spatial-cognitive skills that are foundational to school readiness. Ethnic differences in children’s block-building performance were already seen when children were 4 and 5 years of age; mothers’ and children’s block-building behaviors corresponded in highly specific ways; and mothers’ and children’s block building differed by ethnicity, with U.S. Chinese and Mexican dyads, the most recent immigrant groups, being more likely to emphasize task-specific construction than free-form construction compared to U.S. Dominican and African American dyads.

A first aim was to test ethnic differences in children’s spatial skills based on a block-building assessment. Block building offers children opportunities to manipulate object relations, and has been shown to support later STEM performance in math cognition (Verdine et al., 2017). Chinese children showed higher performance relative to other children even before beginning formal schooling, a finding that mirrors the Asian advantage in early math skill prior to school entry (Sonnenschein and Sun, 2017), and extends work to an informal, yet cognitively important activity in early childhood—building 3D block constructions. Still, within-group variation was striking, with children in every ethnic group ranging from failing most items to mastering the entire set of items. Thus, attention to within-group heterogeneity is critical to any investigation of cultural differences.

When examining mothers’ and children’s block-building interactions, dyads of the four ethnicities did not differ in terms of who initiated and led the block building, although they differed on how mothers and children distributed their time between building task-specific and free-form structures. Mexican and Chinese mothers built more test-specific structures than other mothers, whereas African American and Dominican mothers built more free-form structures. These recent immigrant mothers may have spent relatively more time on test-specific construction because of Mexican mothers’ high endorsement of children’s achievement (Suizzo, 2007) and belief that children learn by following parents’ directions (Keels, 2009), and Chinese mothers’ emphasis on teaching (Huntsinger and Jose, 2009) and view of themselves as active facilitators of children’s learning (Sonnenschein et al., 2018). In contrast, the 3+ generation African American mothers and longer-resident U.S. Dominican mothers may have favored free-form construction because of acculturation to cultural messages around the importance of children’s choice in play and sense of agency (Keller, 2003), and avoidance of drill and practice-oriented teaching methods (Huntsinger and Jose, 2009).

However, Mexican and Chinese mothers diverged in their use of instruction around block building. Although Mexican immigrant mothers used high instruction with their children, Chinese immigrant mothers did not, perhaps because Chinese children already demonstrated high proficiency on block building and needed little further support. In fact, by the time children were 5 years of age, Chinese mothers pulled back from their initially high emphasis on building test-specific structures to building free-form structures with their children, whereas Mexican mothers remained relatively high on test-specific constructions.

A final question concerned whether and how mother-child block building interactions relate to children’s block-building performance. When investigating associations between mother and child block-building behaviors and children’s block-building performance at an *individual* level, beyond ethnicity, mothers’ time spent building test-specific items related to children’s block-building performance at the 4-year assessment specifically, whereas verbal instruction and initiation did not. The association between mothers’ task-specific construction and children’s performance suggests that visually-perceptible, hands-on-guidance by adults may aid children’s block-building skill and understanding of spatial relations more than verbal instruction at young ages. Indeed, how people use their bodies and hands reflects what the mind is doing (Kita et al., 2017); draws children’s attention to where to look and how to act (Zukow-Goldring and Arbib, 2007); and plays a functional role in spatial and mathematical cognition specifically (Hostetter and Alibali, 2019).

Notably, this research contains limitations that suggest promising avenues for future inquiry. First, children’s block-building performance for each test item was coded as correct or incorrect, with no attention to how close children came to succeeding and which types of spatial errors led to failure. Attention to the real-time unfolding of children’s strategies as they work through spatial problems will help inform educational curricula and guide interventions in informal settings such as the home environment.

Second, the session was brief and focused on only one aspect of parent support—mothers’ verbal and physical behaviors during block building in a lab setting. Whether and how parental support for spatial learning manifests in the day-to-day lives of young children remains open to investigation. Indeed, parents’ everyday spatial talk at home (such as naming shapes and referring to spatial dimensions and features), relates to children’s abilities to identify spatial relations in images and mentally transform shapes (Pruden et al., 2011). Furthermore, many factors contribute to what and how parents interact with their children around spatial activities, including parents’ skills, beliefs, anxieties, and so forth.

Third, findings may not generalize to other U. S. Chinese, Mexican, Dominican or African American samples or to populations studied by other researchers. For example, the current sample of recent immigrant Chinese mothers averaged fewer than 11 years of education, which might also explain their lower than expected rates of verbal instruction to children. We are currently expanding focus to children’s spatial skills and everyday experiences around spatial toy play, home literacy, and home numeracy activities as potential contributors to children’s spatial cognitive skills. Additionally, differences in the lexical and grammatical structures of home languages, which varyingly highlight spatial features, relations, and motions (e.g., Choi and Bowerman, 1991; Choi et al., 1999), may contribute to ethnic differences in children’s spatial skills.

The current study provides a first step toward unpacking the potential sources of ethnic and individual differences in children’s early STEM-related experiences and performance. Efforts to educate parents and teachers about the cognitive benefits of block building may go a long way in supporting children’s early spatial skills and thus promoting their math and science understanding. Indeed, play with blocks is compatible with learning rather than a distraction from learning. Elucidating the home environment factors that relate to children’s spatial cognition will help inform parents, educators, and policymakers about ways to support the building blocks for STEM learning in U.S. children from different ethnic, racial, and socioeconomic backgrounds.

## Ethics Statement

This study was carried out in accordance with the recommendations of the University Committee on Activities Involving Human Subjects of New York University with written informed consent from all participants. The protocol was approved by the University Committee on Activities Involving Human Subjects of New York University.

## Author Contributions

EL coordinated the data collection. EL and DS contributed equally to the data analysis. EL led preparation of figures. DS led preparation of the draft of the manuscript. FN aided in the conceptualization, measurement, and design of the study. CT-L supervised the work, provided critical feedback to shape the research design and analysis, and contributed to manuscript writing and revision. All authors read and approved the final manuscript.

## Conflict of Interest Statement

The authors declare that the research was conducted in the absence of any commercial or financial relationships that could be construed as a potential conflict of interest.
